# Learning Reward Uncertainty in the Basal Ganglia

**DOI:** 10.1371/journal.pcbi.1005062

**Published:** 2016-09-02

**Authors:** John G. Mikhael, Rafal Bogacz

**Affiliations:** 1 Department of Experimental Psychology, University of Oxford, Oxford, United Kingdom; 2 Harvard Medical School, Boston, Massachusetts, United States of America; 3 MRC Brain Network Dynamics Unit, University of Oxford, Oxford, United Kingdom; 4 Nuffield Department of Clinical Neurosciences, University of Oxford, Oxford, United Kingdom; George Mason University, UNITED STATES

## Abstract

Learning the reliability of different sources of rewards is critical for making optimal choices. However, despite the existence of detailed theory describing how the expected reward is learned in the basal ganglia, it is not known how reward uncertainty is estimated in these circuits. This paper presents a class of models that encode both the mean reward and the spread of the rewards, the former in the difference between the synaptic weights of D1 and D2 neurons, and the latter in their sum. In the models, the tendency to seek (or avoid) options with variable reward can be controlled by increasing (or decreasing) the tonic level of dopamine. The models are consistent with the physiology of and synaptic plasticity in the basal ganglia, they explain the effects of dopaminergic manipulations on choices involving risks, and they make multiple experimental predictions.

## Author Summary

To maximize their chances for survival, animals need to base their decisions not only on the average consequences of chosen actions, but also on the variability of the rewards resulting from these actions. For example, when an animal’s food reserves are depleted, it should prefer to forage in an area where food is guaranteed over an area where the amount of food is higher on average but variable, thus avoiding the risk of starvation. To implement such policies, animals need to be able to learn about variability of rewards resulting from taking different actions. This paper proposes how such learning may be implemented in a circuit of subcortical nuclei called the basal ganglia. It also suggests how the information about reward uncertainty can be used during decision making, so that animals can make choices that not only maximize expected rewards but also minimize risks.

## Introduction

In situations where actions are associated with rewards, knowledge of the reliability of rewards for alternative choices is critical for selecting the optimal action. Normative models have suggested that optimal foraging requires adaptively switching between risk aversion and risk seeking depending on the circumstance [1, 2]. Indeed, experimental data suggest that humans and animals tend to seek or avoid choice options with reward uncertainty in different situations [1, 3]. To implement such policies, animals and humans need to have estimates of the reward variability associated with different sources, as well as the ability to control how this variability should influence their choices. In addition, knowledge of the reliability of reward feedback is important for learning about the mean reward, as it sets the optimal learning rate. Indeed, in high uncertainty situations, a single new data point should not influence the animal’s previously held estimate as strongly as it would in situations where the uncertainty associated with the data point is fairly low [4]. Furthermore, the estimate of reliability of rewards is helpful in optimizing the exploration-exploitation trade-off [5], because when an animal wishes to find which action yields the highest average reward, it takes more samples to get an accurate estimate of the mean reward for actions with more variable rewards. Hence, such actions should be preferably explored.

One of the key regions of the brain underlying action selection is the basal ganglia (BG). The BG is thought to be involved in learning the expected values of rewards that are associated with given actions and in selecting the actions associated with the highest expected values while inhibiting the others. The learning process in BG is facilitated by neurons releasing dopamine (DA), which encode the reward prediction error, defined as the difference between reward obtained and expected [6, 7]. This signal allows BG to update its estimates of reward accordingly [8, 9].

The pathologies that affect the function of BG influence how it learns or makes decisions in situations involving uncertainty. For instance, a subset of patients with Parkinson’s disease, who suffer from selective death of dopaminergic neurons in the substantia nigra in the midbrain, are impaired in a task involving choices between options with different spreads of their respective reward distributions [10]. When they are on medication (DA agonist), these patients exhibit a well-reported phenomenon of obsessive gambling, in which the patients seem to exhibit a change in their subjective values of risk and reward [11]. This change can be reversed by taking the patients off medication [12]. Additionally, manipulating the levels of dopamine in humans and animals adjusts their decision making under risk [13].

These pieces of evidence suggest that uncertainty is encoded in BG (but one has to note that although BG is the main target of dopaminergic projections, DA neurons also innervate cortex, so some of the effects mentioned above may also have cortical contribution). While computational models have been developed to explain how BG can estimate the expected reward [8, 9, 14, 15], it is still unclear how the reliability of the reward can be estimated in BG, given its anatomical and physiological properties.

Here we show that there exists a class of models consistent with the physiology of BG that can at once learn both the expected reward from a given action and the reliability of the reward, i.e., the spread of its probability distribution. We then show how the models can use learned information about reward uncertainty in decision making, and how the models can account for the effect of dopaminergic medications on decision making in tasks involving risk.

In the next section (“Models”), we review previously proposed models of reinforcement learning in BG, on which our models are built. The new models that can learn reward uncertainty are presented in Section “Results”. Readers familiar with the actor-critic model [16] and Opponent Actor Learning model (OpAL) [15] can skip directly to “Results”.

## Models

The models of reinforcement learning in BG have been developed in two frameworks: a simpler framework considering only an “actor” and a more complex “actor-critic framework.” We review both of these frameworks, as both can be extended to learning reward uncertainty.

### Actor-only framework

This framework assumes that BG estimates average rewards for selecting different actions. Let $Q_{i}^{\left(t\right)}$ denote an estimate of expected reward for selecting the action *i* on trial *t*. Let us assume that after selecting the action, a reward *r*^(*t*)^ is provided, which comes from a distribution with mean *μ*_*i*_ and standard deviation *σ*_*i*_.

We start by considering an abstract Rescorla-Wagner rule [17] for estimating the expected reward for a given action. According to this rule, after receiving a reward, the expected reward is updated in the following way:

$$Q_{i}^{\left(t+1\right)}=Q_{i}^{\left(t\right)}+\alpha \left(r^{\left(t\right)}-Q_{i}^{\left(t\right)}\right)$$ (1)

According to the above equation, the change in the estimate of the expected reward is proportional to the reward prediction error ($r^{\left(t\right)}-Q_{i}^{\left(t\right)}$), scaled by the learning rate constant *α*, where 0 < *α* < 1. It is intuitive to see why this rule works: If *r*^(*t*)^ is underestimated, our estimate *Q*_*i*_ will increase (i.e., $Q_{i}^{\left(t+1\right)}>Q_{i}^{\left(t\right)}$). If *r*^(*n*)^ is overestimated, *Q*_*i*_ will decrease, and if *r*^(*t*)^ is estimated perfectly ($Q_{i}^{\left(t\right)}=r^{\left(t\right)}$), then *Q*_*i*_ will remain the same. In addition, the amount we increment by will be scaled by the magnitude of the prediction error $\left(r^{\left(t\right)}-Q_{i}^{\left(t\right)}\right)$, so that we learn more quickly when we have a lot of learning to do than when our estimate is quite close to the true mean already. Also note that having *α* < 1 ensures that the new data point updates our estimate but does not completely replace it (as would be the case if *α* were in fact equal to 1), an implicit acknowledgement of the existence of uncertainty in the reward and noise in the system.

### Actor-critic framework

The actor-critic model [16] includes two components: an actor that learns tendencies to select particular actions and a critic that learns an overall value of the current context or state. In the actor-critic model, the value *V* of being in this state is learned by the critic according to the standard Rescorla-Wagner rule [17] (cf. Eq 1):

$$V^{\left(t+1\right)}=V^{\left(t\right)}+\alpha \left(r^{\left(t\right)}-V^{\left(t\right)}\right)$$ (2)

Note that *V*^(*t*)^ is updated regardless of which action *i* is selected, so *V*^(*t*)^ is not an estimate of expected reward associated with a particular action, but rather an average reward in the current state.

In the standard actor-critic model, after choosing action *i*, the tendency to choose it, which we denote by *Q*_*i*_, is learned by the actor using the following update rule:

$$Q_{i}^{\left(t+1\right)}=Q_{i}^{\left(t\right)}+\alpha \left(r^{\left(t\right)}-V^{\left(t\right)}\right)$$ (3)

According to the above equation, the tendency to choose action *i* is also modified proportionally to the reward prediction error, i.e., it is increased if the action resulted in a higher reward than expected by the critic and decreased if the reward was below expectation.

**Fig 1:**
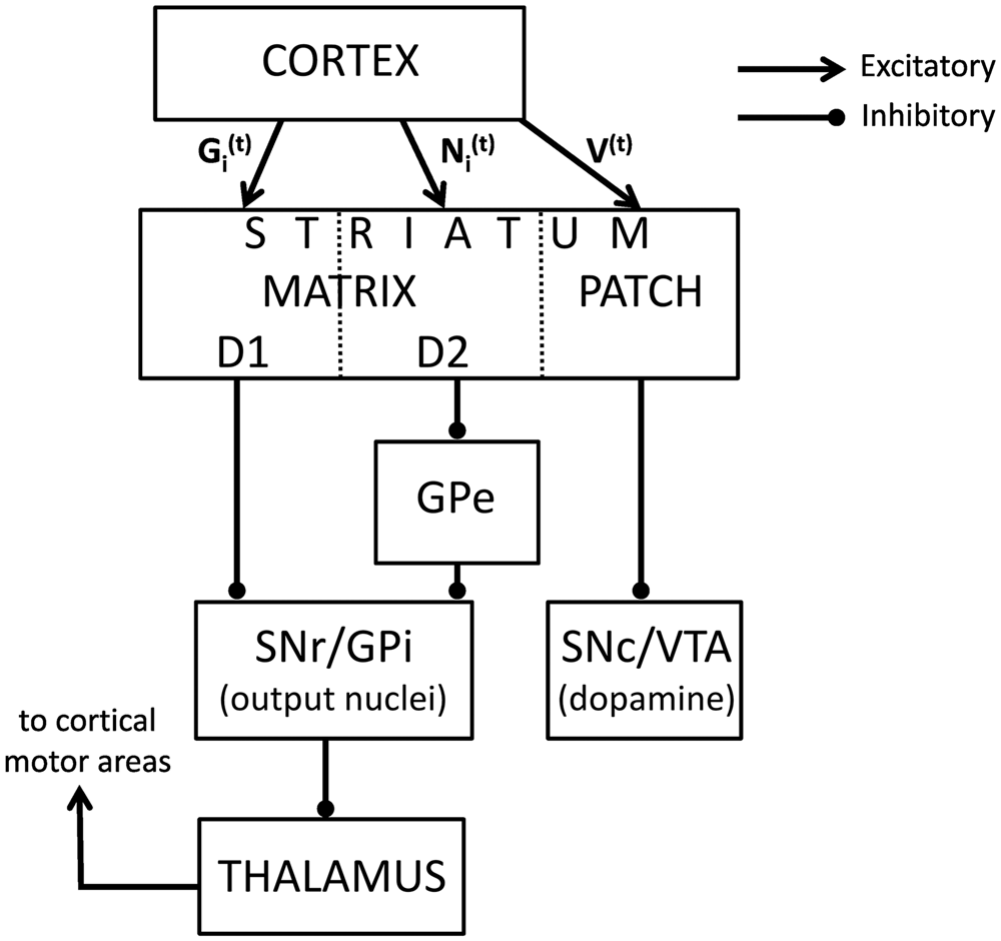
Simplified anatomy of the basal ganglia. The arrows and lines ending with circles denote the excitatory and inhibitory connections respectively. The following abbreviations are used: GPe—external globus pallidus, SNr—substantia nigra pars reticulata, GPi—internal globus pallidus, SNc—substantia nigra pars compacta, VTA—ventral tegmental area.

The actor-critic model naturally maps on the matrix-patch organization of the striatum [18]. Such mapping assumes that *V*^(*t*)^ is encoded in the synapses between cortical neurons selective for the current context and striatal patch neurons, as shown in Fig 1. The patch neurons directly inhibit dopaminergic neurons [19], so that if the dopaminergic neurons also receive input encoding reward, then their activity may encode *r*^(*t*)^ − *V*^(*t*)^. The actor part of the model is mapped on matrix neurons [18] that send projections to the output nuclei, which in turn project to areas controlling movement, so they can affect which movement is selected. Finally, the dopaminergic neurons modulate plasticity of the synapses of both patch neurons and matrix neurons. It is worth adding that some studies map actor and critic on dorsal and ventral striatum respectively [20], but this mapping is related to the matrix-patch mapping, as the patch neurons are more common in ventral than dorsal striatum [21].

### Opponent actor learning model

A recent model called Opponent Actor Learning (OpAL) [15] takes into account the fact that the matrix neurons can be subdivided into two groups, which express D1 and D2 DA receptors, respectively. These project through different nuclei of BG, as shown in Fig 1 [22] and have opposite effects on movement initiation [23, 24]. In particular, D1 neurons project through the “direct” pathway to the output nuclei, and their activity facilitates movements [25] because they inhibit the output nuclei and thus release thalamus from inhibition. By contrast, D2 neurons project through the “indirect” pathway, and their activity inhibits movement [25].

The OpAL model describes learning about the tendencies to choose or inhibit actions *i* in a given state, which we will denote by $G_{i}^{\left(t\right)}$ (for Go) and $N_{i}^{\left(t\right)}$ (for NoGo), respectively. The OpAL model proposes that these tendencies are encoded in the strengths of synaptic connections between the cortical neurons associated with that state and the striatal D1 or D2 neurons selective for action *i*, respectively [15], as illustrated in Fig 1. In the OpAL model, after selecting action *i* the synaptic weights are modified according to:

$$G_{i}^{\left(t+1\right)}=G_{i}^{\left(t\right)}+\alpha G_{i}^{\left(t\right)}\left(r^{\left(t\right)}-V^{\left(t\right)}\right)$$ (4)

$$N_{i}^{\left(t+1\right)}=N_{i}^{\left(t\right)}-\alpha N_{i}^{\left(t\right)}\left(r^{\left(t\right)}-V^{\left(t\right)}\right)$$ (5)

Thus if the reward prediction error is positive, the tendency to select the action is increased, while the tendency to inhibit it is weakened, and vice versa. Additionally, in the OpAL model, the reward prediction error is scaled by $G_{i}^{\left(t\right)}$ and $N_{i}^{\left(t\right)}$, which prevents $G_{i}^{\left(t\right)}$ and $N_{i}^{\left(t\right)}$ from becoming negative. For example, if $G_{i}^{\left(t\right)}$ becomes close to 0, the changes in its value also tend to 0.

The OpAL model additionally proposes how the probabilities of actions depend on the weights in Go and NoGo pathways, through a generalized version of the softmax rule [26, 27]:

$$P_{i}^{\left(t\right)}=\frac{\text{exp}\left(aG_{i}^{\left(t\right)}-bN_{i}^{\left(t\right)}\right)}{\sum_{k}\text{exp}\left(aG_{k}^{\left(t\right)}-bN_{k}^{\left(t\right)}\right)}$$ (6)

In the above equation, normalization by the denominator ensures that the $P_{i}^{\left(t\right)}$ add up to 1 across all possible actions. Parameters *a* and *b* control how deterministic the choice is: when *a* = *b* = 0, all actions have equal probability, while with higher *a* and *b*, the influence of the learned tendencies on choice increases. The relative value of parameters *a* and *b* describes to what extent the neurons in the Go and NoGo pathways contribute to choice (when *a* = *b*, both pathways contribute equally; otherwise, one pathway dominates). The rationale for introducing two parameters *a* and *b* is that the activity levels of the striatal D1 and D2 neurons are modulated in opposite directions by levels of DA; hence, they can differentially contribute to activity in the output nuclei [15] (see Fig 1).

## Results

We first describe the conceptually simpler actor-only model, which will allow for a clearer explanation of the essential mechanisms of learning reward uncertainty. Then, we show how the model can explain the effect of dopaminergic stimulation on choice in tasks involving selection between safe and risky options, Subsequently, we present generalizations of the model, and compare it with the OpAL model.

### Learning reward uncertainty in actor-only framework

In the models including only the actor, learning about the reward distribution of an individual action is independent of learning about the distribution of another. Thus for simplicity of notation, while introducing the model we will consider just a single context and a single action, and denote the corresponding synaptic weights of D1 and D2 neurons on trial *t* by *G*^(*t*)^ and *N*^(*t*)^, respectively. Furthermore, we will denote the mean and standard deviation of reward distribution by *μ*_*r*_ and *σ*_*r*_.

The model employing the original Rescorla-Wagner rule (Eq 1) keeps track of an abstract variable *Q*^(*t*)^ that describes the overall tendency to select action *i*, but in BG this tendency is encoded in the synaptic weights of D1 and D2 neurons, *G*^(*t*)^ and *N*^(*t*)^. So let us relate these variables by:

$$Q^{\left(t\right)}=G^{\left(t\right)}-N^{\left(t\right)}$$ (7)

The update rules for the weights in the Actor learning Uncertainty (AU) model have the following form:

$$G^{\left(t+1\right)}=G^{\left(t\right)}+\alpha {\left|r^{\left(t\right)}-Q^{\left(t\right)}\right|}_{+}-\beta G^{\left(t\right)}$$ (8)

$$N^{\left(t+1\right)}=N^{\left(t\right)}+\alpha {\left|r^{\left(t\right)}-Q^{\left(t\right)}\right|}_{-}-\beta N^{\left(t\right)}$$ (9)

In the equations above, the prediction errors are transformed through threshold-linear functions |*x*|_+_ and |*x*|_−_ which are equal to |*x*| if *x* is positive or negative respectively, and 0 otherwise. In other words, |*x*|_+_ = max(*x*, 0), and |*x*|_−_ = max(−*x*, 0). Thus if the prediction error is positive, then so is the corresponding term in Eq (8), and *G* increases, while if the prediction error is negative, then the corresponding term in Eq (9) is positive, and *N* increases. Furthermore, the decay terms (last terms in Eqs (8) and (9)) are scaled by a separate constant 0 < *β* < 1.

As we will explain below, the AU model encodes the estimate of mean reward *μ*_*r*_ in *G*^(*t*)^ − *N*^(*t*)^, while the estimate of reward spread *σ*_*r*_ in *G*^(*t*)^ + *N*^(*t*)^. Before giving a proof for this property, let us first provide an intuition. The AU model encodes the mean reward in *G*^(*t*)^ − *N*^(*t*)^ due to its similarity with the Rescorla-Wagner rule. In particular, when the reward is higher than expected, *G* tends to increase, while when the reward is lower than expected, *N* tends to increase, so in both cases *G*^(*t*)^ − *N*^(*t*)^ tends to move towards the value of the reward.

**Fig 2:**
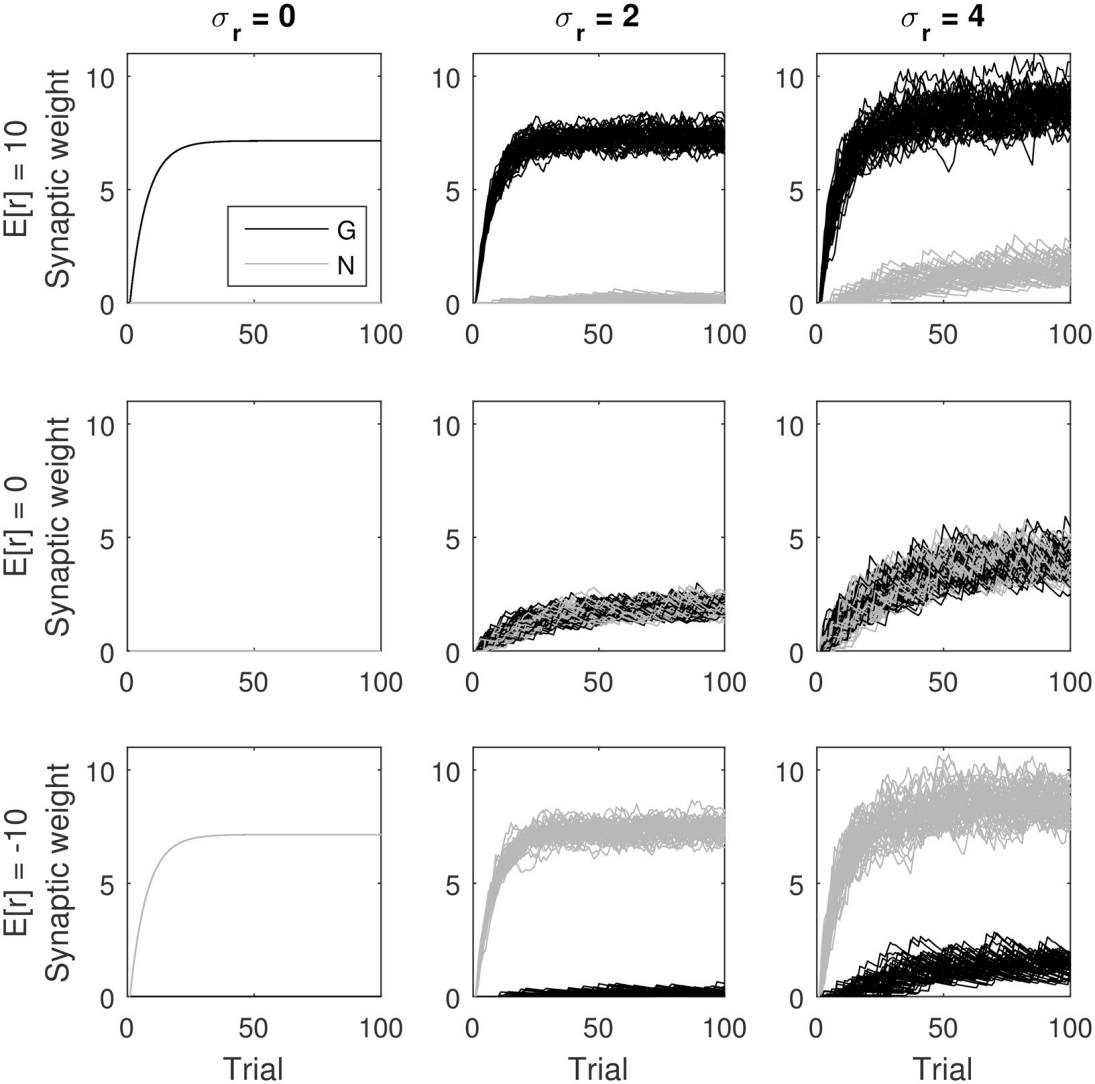
Changes in *G* and *N* for the AU model as a function of trial number. Different rows correspond to different mean reward *μ*_*r*_ (indicated left of each row), and different columns correspond to different standard deviations of reward *σ*_*r*_ (indicated above each column). The rewards were sampled from a Gaussian distribution. Here, both *G* and *N* were initialized at 0, and we set *α* = 0.1. We have selected $\beta =\frac{\alpha }{\sqrt{2\pi }}$ in order to make the figure easier to interpret, because then $\frac{\alpha }{2\beta }E(|r-\mu_{r}\left|\right)=\sigma_{r}$, and hence in the middle row *G* and *N* approach *σ*_*r*_. For each of the panels, the simulation was run 50 times, for 100 trials each.

To gain some intuition for how the model can encode reward uncertainty in *G*^(*t*)^ + *N*^(*t*)^, it is useful to consider the changes in the weights in two different cases: when the rewards are deterministic, i.e., of the same magnitude each time the action is selected, and when they are stochastic. In the case of deterministic rewards, on initial trials, reward prediction error will be positive, hence only *G* will increase but not *N*, as illustrated in the top left panel of Fig 2. By contrast, in the case of stochastic rewards, on some trials the reward prediction error will be negative. Hence, *N* will also increase, as illustrated in the top right panel of Fig 2.

Finally, the decay terms in the above equations serve to ensure the convergence of the synaptic weights, as in their absence, the update rules would only allow *G* and *N* to either increase or stay the same upon every iteration, but never decrease.

Let us now show that the AU model can learn expected reward. By subtracting Eq (9) from Eq (8) we obtain:

$$Q^{\left(t+1\right)}=Q^{\left(t\right)}+\alpha \left(r^{\left(t\right)}-Q^{\left(t\right)}\right)-\beta Q^{\left(t\right)}$$ (10)

The threshold-linear functions disappear when Eqs (8) and (9) are subtracted, because if the prediction error is positive, the corresponding terms in Eqs (8) and (9) are equal to the prediction error and 0 respectively, so when subtracted give the prediction error. Conversely, if the prediction error is negative, the corresponding terms in Eqs (8) and (9) are equal to 0 and the negative of the prediction error, so when subtracted they also give the prediction error. Comparing Eqs (10) and (1), we note that this update rule is similar to the standard Rescorla-Wagner rule, with an added decay term.

For a fixed value of *α*, the variable *Q* never converges when *σ*_*r*_ > 0, but constantly fluctuates. Nevertheless, it is useful to consider a value around which it fluctuates. After sufficiently long learning, the expected change in *Q* will be zero. In other words, for large enough *t*,

$$E [Q^{\left(t+1\right)}-Q^{\left(t\right)}]=0$$ (11)

The value of *Q*^(*t*)^ at which Eq (11) holds is referred to as the stochastic fixed point, and we will denote it by $Q_{i}^{*}$. By combining Eq (10) with Eq (11), we obtain:

$$E [\alpha \left(r-Q^{*}\right)-\beta Q^{*}]=0$$ (12)

Rearranging the terms in the above equation, we see that *Q* at the stochastic fixed point is equal to:

$$Q^{*}=\frac{\alpha }{\alpha +\beta }E\left[r\right]$$ (13)

Although in the AU model Q* is not equal to the expected reward, it is proportional to it, with a proportionality constant that is equal across all actions. Thus, choosing an action with the highest Q* is equivalent to choosing an action with the highest expected reward.

We now show that the AU model learns reward uncertainty. In order to do so, we will analyze how the sum of the synaptic weights evolves. Thus, let us define:

$$S^{\left(t\right)}=G^{\left(t\right)}+N^{\left(t\right)}$$ (14)

By adding Eq (9) to Eq (8):

$$S^{\left(t+1\right)}=S^{\left(t\right)}+\alpha \left|r^{\left(t\right)}-Q^{\left(t\right)}\right|-\beta S^{\left(t\right)}$$ (15)

From the above equation we see that at the stochastic fixed point:

$$\begin{array}{ll} S^{*} & =\frac{\alpha }{\beta }E\;[\left|r-Q^{*}\right|\text{]}\\ & =\frac{\alpha }{\beta }E\;[\left|r-\frac{\alpha }{\alpha +\beta }\mu_{r}\right|\text{]} \end{array}$$ (16)

The above equation implies that when Q* = *μ*_*r*_, the sum of *G* and *N* is equal to the deviation of the reward from the mean. In S1 Text we illustrate that, when Q* = *μ*_*r*_, then S* is directly proportional to the standard deviation or variance of rewards (depending on the shape of the reward distribution). When Q* ≠ *μ*_*r*_, S* is not exactly proportional to the deviation of the rewards from the mean. To see more clearly when it approximates the deviation, let us rewrite the above equation as:

$$S^{*}=\frac{\alpha }{\beta }E [\left|\left(r-\mu_{r}\right)+\frac{1}{\frac{\alpha }{\beta }+1}\mu_{r}\right|]$$ (17)

From the equation above, we see that S* becomes proportional to the deviation of rewards when the second term inside the expected value is dominated by the first. This can occur in two cases. First, since the magnitude of the first term increases with *σ*_*r*_, while that of the second term is proportional to *μ*_*r*_, then *S* is close to an estimate of the deviation of rewards when *σ*_*r*_ is relatively high with respect to *μ*_*r*_.

Fig 2 shows simulations of the model for different reward mean and standard deviations of rewards and illustrates changes in synaptic weights as learning progresses. The simulations shown in different rows correspond to mean reward being positive, equal to 0, and negative, respectively. Note that the difference between *G* and *N* always approaches a value proportional to the expected reward. The simulations shown in different columns correspond to progressively higher standard deviation of reward. When *μ*_*r*_ = 0, the value that *G* and *N* approach increases linearly with *σ*_*r*_. By contrast, when *μ*_*r*_ is higher, the encoding of reward uncertainty is less precise. For example, in the top row of Fig 2 we observe that the values of synaptic weights change very little as *σ*_*r*_ increases from 0 to 2. The increase in weights is slightly higher as *σ*_*r*_ increases from 2 to 4. Nevertheless, Fig 2 shows that increasing reward uncertainty still results in higher values of both *G* and *N*. Note that in each row, the larger the reward uncertainty, the larger *G* and *N*.

**Fig 3:**
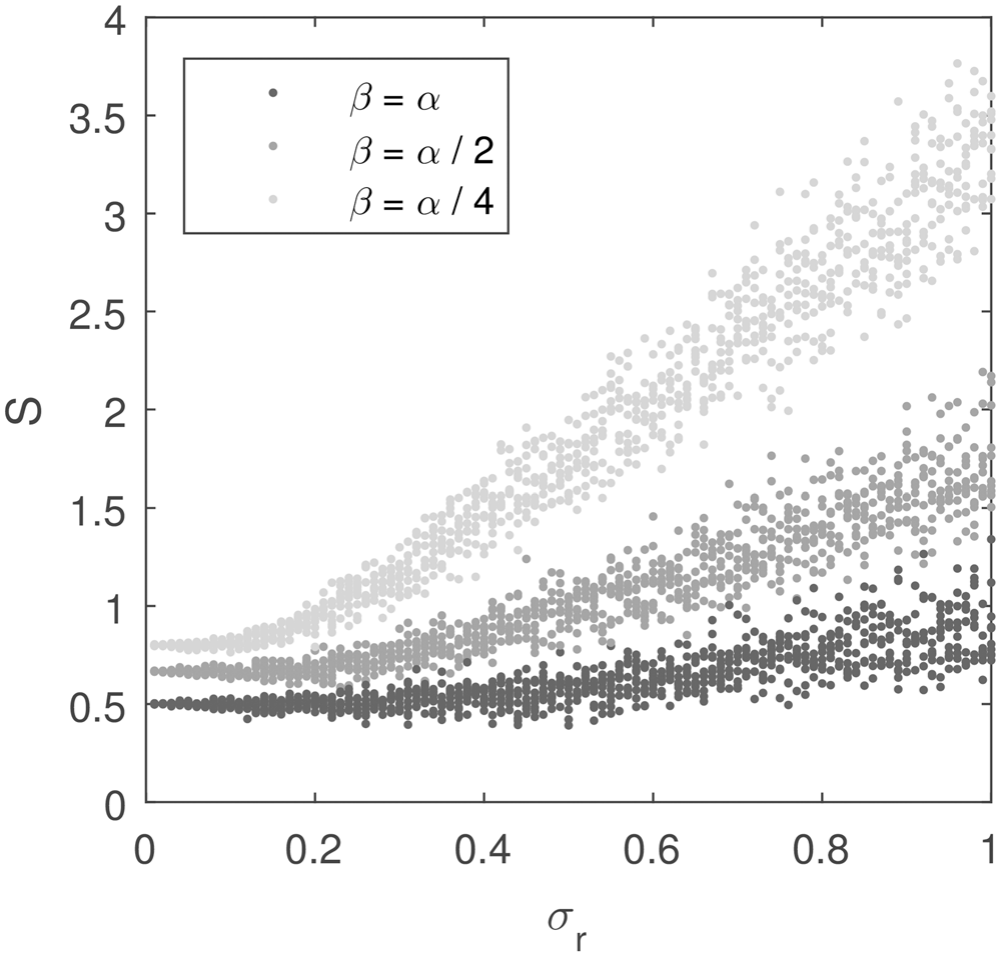
Comparison of the sum of weights in the Go and NoGo pathways in the AU model (vertical axis) with the standard deviation of rewards (horizontal axis) for different values of parameter *β*. In all simulations in this figure, *μ*_*r*_ = 1 (so *σ*_*r*_ is equal to the coefficient of variation) and *α* = 0.1. For each value of *σ*_*r*_ the model was simulated 10 times for 300 iterations. For each simulation, the sum of *G* and *N* at the end of the simulation is displayed as a point on the figure.

Second, the second term in Eq (17) decreases with the ratio of parameters $\frac{\beta }{\alpha }$. Thus the lower *β* is relative to *α*, the closer S* is to a linear function of the deviation of rewards. This property is illustrated in Fig 3, which plots *S* as a function of the standard deviation of rewards for different values of *β*. It is evident in the figure that, on average, *S* is a monotonic function of *σ*_*r*_. Hence, it is worth noting that although *S* is an estimate of reward uncertainty, it is possible for the neural system to obtain a closer estimate by learning the function mentioned above and thus decode the estimate of reward deviation from *S* (i.e., correct the biases of *S* in estimating *σ*_*r*_). However, this function has a flat region for low *σ*_*r*_, so that the model’s estimate of the reward deviation will not be precise in that range of *σ*_*r*_. For example, one can observe in Fig 3 that when *β* = *α* the value of *S* ≈ 0.5 arises for a wide range of *σ*_*r*_, so knowing that *S* = 0.5 we cannot accurately tell the value of *σ*_*r*_. The size of the region where *σ*_*r*_ is not well estimated can be reduced by decreasing *β* relative to *α*. Nevertheless, Fig 3 illustrates that there is a trade-off: Lower $\frac{\beta }{\alpha }$ results in a higher magnitude of weights, and thus higher metabolic cost, and lower *β* also slows learning (see [28] for details).

### Control over risk seeking via dopamine level

Let us now consider how the mean and spread of a reward distribution, learned by the model described above, can be used by BG in action selection. In the model the tendency to choose or avoid risky options is controlled by the tonic level of DA. Before giving mathematical justification for this property, let us first provide an intuition for it.

**Fig 4:**
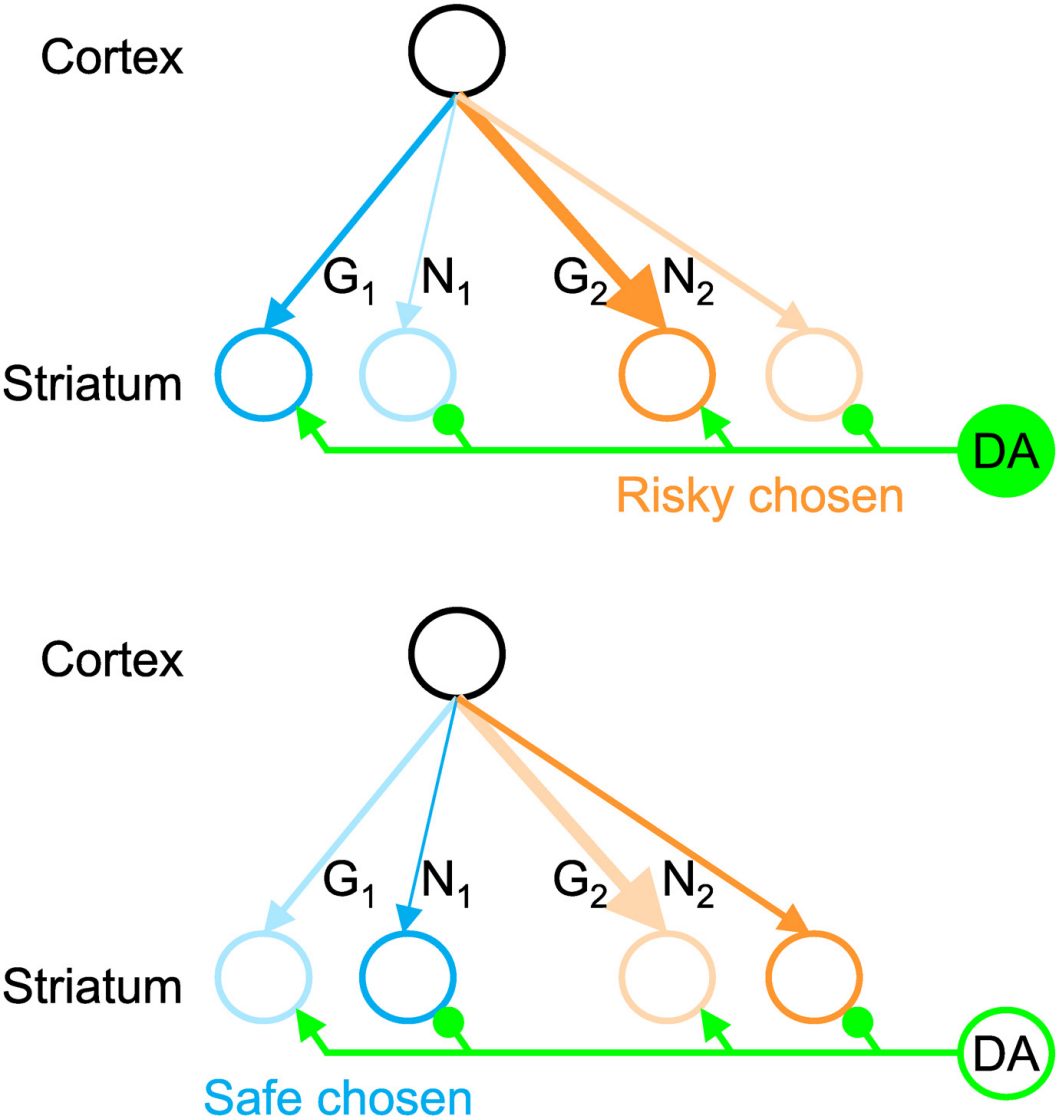
Effects of dopamine (DA) on action selection between safe and risky options. Circles denote different neural populations: black circle corresponds to the neural population in cortex selective for the current state, green circle corresponds to dopaminergic neurons, and blue and orange circles correspond to the striatal neurons selective for two different actions. The circles receiving inputs via connections *G*_*i*_ and *N*_*i*_ correspond to D1 and D2 neurons. Arrows and lines ending with circles denote connections with excitatory and inhibitory effect respectively. The top panel illustrates a situation of high tonic level of DA, where the D2 neurons are inhibited (indicated by bleak color), while the bottom panel corresponds to low DA, where the D1 neurons are inhibited.

Fig 4 illustrates states of a network choosing between two options, one safe and the other risky, represented by neurons shown in blue and orange, respectively. In the figure, the strength of cortico-striatal connections is denoted by the thickness of the arrows. Thus both options are associated with positive mean reward (as the connections *G*_*i*_ are thicker than *N*_*i*_), but the orange option has higher estimated spread of rewards (as the orange connections are thicker than the blue ones). DA is known to activate the D1 or Go neurons and inhibit D2 or NoGo neurons, which is represented in Fig 4 by green arrows and lines ending with circles. The top panel illustrates a situation when the tonic DA level is high. In this case the NoGo neurons are suppressed (indicated by bleak color) and the choice is driven by the activity of the Go neurons. Thus with high DA, the more risky, orange option is more likely to be chosen, as *G*_2_ > *G*_1_. By contrast, with low levels of DA, the Go neurons are inhibited (bottom panel of Fig 4), and the choice is driven by NoGo neurons. Thus with low DA, the risky option is inhibited (as *N*_2_ > *N*_1_), and the model is more likely to select the safe option.

The above example illustrates that the model has the tendency to choose more risky options when the level of DA is high, and safer options otherwise. Let us now show this property formally. The choice rule of Eq (6) can be rewritten to make the effect of the mean and deviation of reward visible. To do so, we first write *G*_*i*_ and *N*_*i*_ in terms of *Q*_*i*_ and *S*_*i*_ (defined in Eqs (7) and (14)):

$$G_{i}^{\left(t\right)}=\frac{1}{2}\left(S_{i}^{\left(t\right)}+Q_{i}^{\left(t\right)}\right)$$ (18)

$$N_{i}^{\left(t\right)}=\frac{1}{2}\left(S_{i}^{\left(t\right)}-Q_{i}^{\left(t\right)}\right)$$ (19)

Substituting the above into Eq (6) we obtain:

$$P_{i}^{\left(t\right)}=\frac{\text{exp}\left(\frac{1}{2}U_{i}\right)}{\sum_{k}\text{exp}\left(\frac{1}{2}U_{k}\right)},\;\;\;\text{where}\;\;\;U_{i}=\left(a+b\right)Q_{i}^{\left(t\right)}-\left(b-a\right)S_{i}^{\left(t\right)}$$ (20)

In the choice rule above, the probability of choice depends on a utility function *U*_*i*_ that is a linear combination of mean reward and the deviation of reward (cf. [29, 30]). By increasing *b* relative to *a* in the above choice rule, one can explicitly control how choice probability is affected by the deviation of rewards. In particular, when *b* > *a*, the uncertainty of rewards reduces the probability of selecting the corresponding action, resulting in risk aversion. By contrast, setting *b* < *a* increases the probability of choosing actions with uncertain rewards, resulting in risk seeking.

Recall that parameters *a* and *b* describe in the OpAL model [15] to what extent D1 and D2 neurons contribute to determining choice. Since high levels of DA activate the direct pathway and suppress the indirect pathway, increasing the tonic level of DA will correspond in the model to increasing *a* and decreasing *b*, which according to the analysis above would result in more risk-seeking behavior. Thus such modulation provides a mean by which an organism can control whether the action selection should be risk-averse or risk-seeking. The above analysis explains why a tendency for gambling in Parkinson’s patients [12, 31] may arise from increasing the level of DA by medications or from deep brain stimulation of subthalamic nucleus (which would also weaken the indirect pathway so would correspond to lowering *b*).

**Fig 5:**
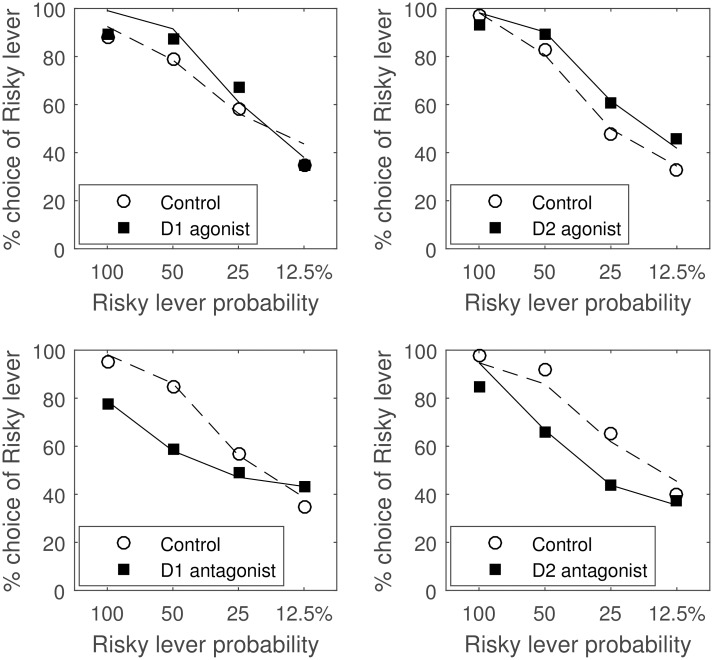
Effect of dopaminergic receptor manipulations on risky choices. In each panel the percentage of risky choices is plotted against the probability of obtaining the large reward by choosing the risky lever. Open circles show the data from animals in the control state, while filled squares show the data obtained after treatment with a drug. Each panel corresponds to a different drug indicated in the key. The data in each panel were read from one figure in [32] and averaged over different drug concentrations. In particular, the data in the four panels come from Figs 3c, 4c, 3a and 4a in [32]. Solid and dashed curves show the fractions of risky choices made by the model, simulated for parameters corresponding to control and drug conditions. During each simulation the model made 10,000 choices in each of four probability conditions (thus the standard error of mean fraction of risky choices made by the model was < 1%). This large number of simulated trials allowed the model to produce stable behavior, which was necessary for the search for parameters resulting in a match with animal behavior.

The presented model accounts for the effect of pharmacological manipulations affecting dopaminergic receptors on risk aversion in reinforcement learning tasks. In a particularly comprehensive study [32], rats were trained to choose between 2 levers: pressing one of them resulted in certain delivery of a single food pellet, while pressing another could result either in delivery of 4 pellets or none. The probability of receiving the large reward after the selection of the risky lever was varied across conditions. After the rats were well-trained in the task, they were injected with different drugs, and changes in the fraction of risky choices made were measured. An overall increased tendency to choose the risky option was observed either after injection of D1 agonist or D2 agonist, as shown in Fig 5. Furthermore, the injection of D1 antagonist or D2 antagonist decreased the tendency to choose the more risky option [32].

The fraction of risky choices made in simulations by the AU model is shown by curves in Fig 5. In the simulations, the parameters controlling learning were fixed to standard values (*α* = *β* = 0.1), and only the parameters controlling choice (*a* and *b*) were fit to the data. Parameters *a* and *b* were fit separately to the data in each panel of Fig 5, as each panel was obtained from a different group of rats. While fitting the model to the data from D1 receptor manipulations, it was assumed that *a* differed between control and drug conditions, while *b* did not change. Thus three parameters were fit: *a*_*control*_, *a*_*drug*_, and *b*. We did not enforce any relationship between *a*_*control*_ and *a*_*drug*_, but as we will explain below, the estimated parameters followed the relationship expected from the known effects of drugs. Analogously, while fitting the model to the data from D2 receptor manipulations, *a*, *b*_*control*_, and *b*_*drug*_ were fit. For each panel, the values of the three parameters were found that minimized the sum of squared errors between the fraction of risky choices made by the animals and the model in the 8 conditions (4 probabilities of large rewards on and off the drug). The parameters were found using the simplex algorithm [33] implemented in Matlab (function fminsearch). The search was repeated 10 times with different random initial parameter values sampled from the range [0, 3].

The model reproduced the fractions of risky choices made by the animals relatively well. Importantly, the overall direction of changes in risky choices and estimated parameters is consistent with the pattern in the data. In particular, in the top panels of Fig 5, the fraction of risky choices is higher in the simulation of the agonist conditions. Furthermore, in the top left panel, estimated parameters satisfied *a*_*drug*_ > *a*_*control*_ (*a*_*control*_ = 1.71, *a*_*drug*_ = 3.13, *b* = 0.59), which is consistent with the excitatory effect of DA on D1 receptors, while in the top right panel, the estimated parameters satisfied *b*_*drug*_ < *b*_*control*_ (*a* = 2.72, *b*_*control*_ = 1.86, *b*_*drug*_ = 0.39), consistent with the inhibitory effect of DA on D2 receptors. Thus the choice behavior may become more risky due to activation of either D1 or D2 receptors, as activation of either of them decreases *b* − *a*, which reduces risk aversion in Eq 20. Analogously in the bottom panels of Fig 5, the fraction of risky choices is lower in the simulated condition with antagonists, and estimated parameters satisfy *a*_*drug*_ < *a*_*control*_ for the bottom left (*a*_*control*_ = 2.67, *a*_*drug*_ = 0.86, *b* = 1.04) and *b*_*drug*_ > *b*_*control*_ for the bottom right panels (*a* = 1.95, *b*_*control*_ = 0.04, *b*_*drug*_ = 2.16).

It is worth noting in the bottom left panel of Fig 5 that the model reproduces the cross-over of the two curves. It occurs in the simulations because as *a* is reduced (corresponding to the effect of D1 antagonist), the choice in the model becomes more random (recall from the Models section that *a* and *b* also control how deterministic the choice is), so that the fraction of risky choices is closer to 50%. In this task, choosing the risky lever gave higher expected reward in the 100% and 50% conditions while choosing the safe lever had higher mean reward in the 12.5% condition, and the model simulated with higher *a* in the bottom left panel of Fig 5 exploited the options with higher expected rewards more.

### Relationship of the model to synaptic plasticity in the striatum

The AU model assumes particular rules for updating striatal synaptic weights, and here we consider whether these rules are consistent with the existing data concerning synaptic plasticity in the striatum. For a synaptic plasticity rule to be plausible, the change in a synaptic weight needs to depend only on the information that can be sensed by a synapse, i.e., the activity of pre-synaptic and post-synaptic neurons, the levels of neuromodulators released in the vicinity of the synapse, and the synaptic weight itself. Eqs (8) and (9) describe the change in synaptic weights between the neurons encoding current context and those encoding current movement, i.e., they describe changes in synapses between co-active neurons. This change includes two terms, which are the reward prediction error and decay. As mentioned earlier, a plethora of evidence suggests that reward prediction error (*r*^(*t*)^ − *Q*^(*t*)^) is encoded in phasic changes in DA concentration, which is released in striatum.

The proposed weight update rules are consistent with the pattern of synaptic plasticity modulation by DA [34]. It has been observed experimentally that the activation of cortical neurons followed by striatal D1 neurons strengthens the synapses of D1 neurons when the DA level is elevated, and weakens these synapses when the DA level is reduced (Figs 3F and 2E in [34]). Such changes are consistent with Eq (8), because for positive prediction error, the prediction error term will dominate, so *G* will increase. By contrast, if the prediction error is negative, |*r*^(*t*)^ − *Q*^(*t*)^|_+_ will be equal to 0, and the decay term will dominate, so *G* will decrease. Conversely, the activation of cortical neurons followed by striatal D2 neurons weakens the synapses of D2 neurons when the DA level is elevated, and strengthens the synapses of D2 neurons when DA level is reduced (Figs 1H and 3B in [34]). Such changes are consistent with Eq (9) for analogous reasons.

A critical property of the learning rules allowing encoding reward uncertainty in *G*^(*t*)^ + *N*^(*t*)^ is the asymmetry in how synaptic weights change for positive and negative reward prediction error. In particular, in the AU model, the change in *G* is only proportional to the reward prediction error if the error is positive, but not if the error is negative (analogous asymmetry holds for *N*). It is easy to check that if such asymmetry were not present (i.e., nonlinear functions of predictions errors were removed from Eqs (8) and (9)), then *G*^(*t*)^ + *N*^(*t*)^ would no longer encode the spread of reward distribution.

**Fig 6:**
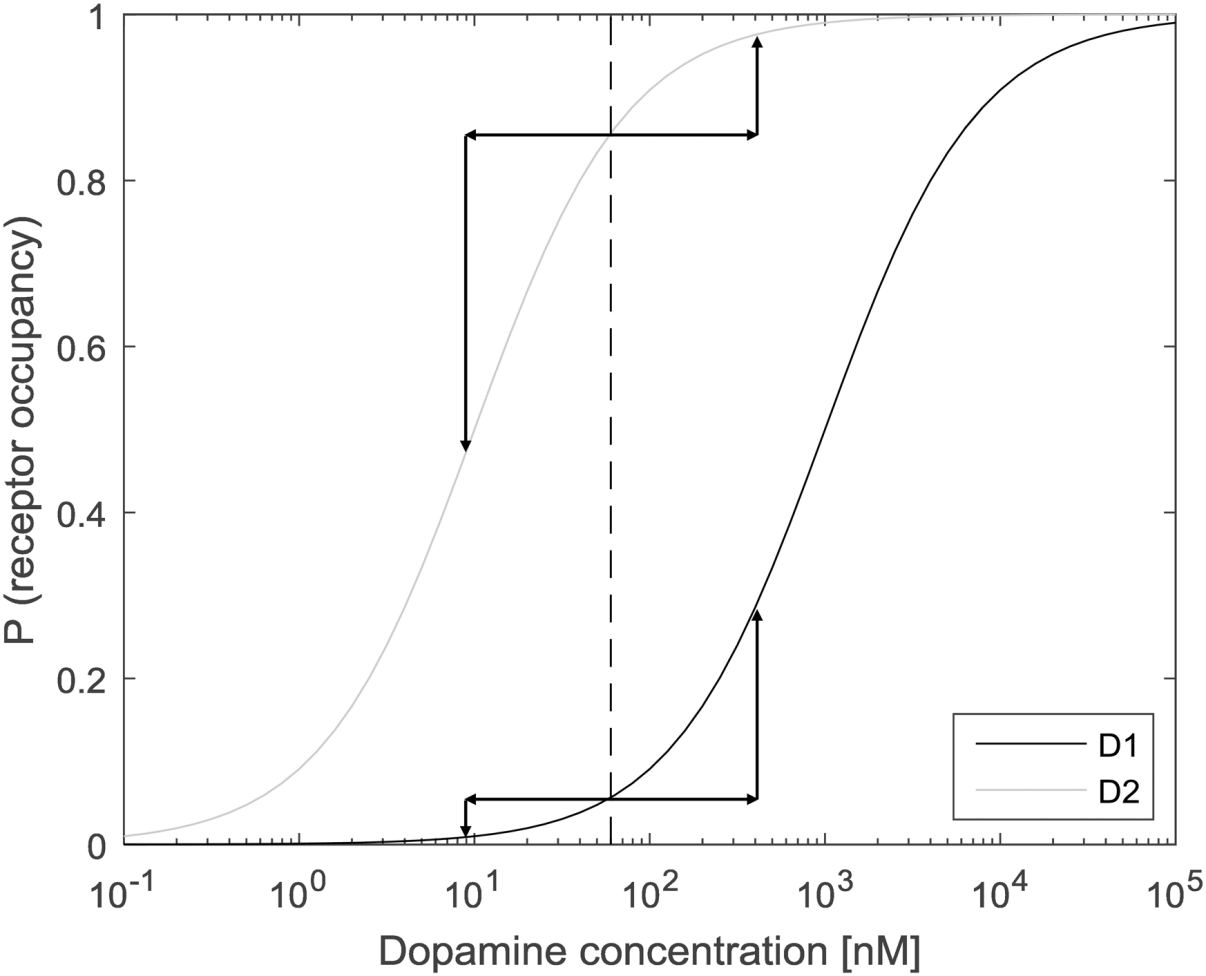
Schematic illustration of the sensitivity of D1 and D2 receptors to the changes in dopamine (DA) level. Black and grey curves show how the probability of D1 and D2 receptor occupancy depends on DA concentration in a biophysical model of [36]. They assumed that receptor occupancy depends on DA concentration *C* as $\frac{C}{EC_{50}+C}$, where *EC*_50_ is the receptor affinity, which was taken as 1*μM* and 10*nM* for D1 and D2 receptors respectively, based on [38]. Dashed line indicates baseline DA concentration *C* = 60*nM* suggested by simulations in [37]. Vertical arrows indicate how much binding probability changes due to changes in DA concentration, shown by horizontal arrows.

Such asymmetry may arise in striatal synapses from the observed differences in the affinity of DA receptors, such that a higher DA concentration is necessary to activate D1 receptors than D2 receptors [35]. Fig 6 shows how the probability of D1 and D2 receptor activation depends on DA concentration in a biophysically realistic model of DA release [36]. Simulation of that model based on activity of DA neurons in vivo [37] suggested that the baseline DA level in striatum is in a sensitive range of both D1 and D2 receptors (as illustrated by the dashed line in Fig 6). Due to the arrangement shown in Fig 6, an increase in DA level has a larger effect on the activation of D1, while a decrease in DA has a larger effect on D2 receptors.

According to Fig 6, the decrease in DA level may still have some small effect on the binding probability of D1 receptors (analogously the increase in DA may have a small effect on D2 receptors). Hence the complete lack of effect of a decrease (increase) in DA level on D1 (D2) neurons’ plasticity may seem inconsistent with the above analysis. Nevertheless below we show that for learning reward uncertainty, it is sufficient that there exist an asymmetry in the dopaminergic effects on the receptors, i.e., that the increase in DA level affect plasticity of D1 neurons more than D2 neurons (and the opposite for a decrease in DA level).

The Equations describing the AU model can be generalized to include more complex functions of reward prediction error:

$$G^{\left(t+1\right)}=G^{\left(t\right)}+\alpha \left({\left|r^{\left(t\right)}-Q^{\left(t\right)}\right|}_{+}-\epsilon {\left|r^{\left(t\right)}-Q^{\left(t\right)}\right|}_{-}\right)-\beta G^{\left(t\right)}$$ (21)

$$N^{\left(t+1\right)}=N^{\left(t\right)}+\alpha \left({\left|r^{\left(t\right)}-Q^{\left(t\right)}\right|}_{-}-\epsilon {\left|r^{\left(t\right)}-Q^{\left(t\right)}\right|}_{+}\right)-\beta N^{\left(t\right)}$$ (22)

where *ϵ* is a constant such that *ϵ* < 1. As synaptic weights cannot be negative, whenever *G*^(*t*+1)^ or *N*^(*t*+1)^ computed from the above equations is negative, it is set to 0. A potential advantage of using such functions of prediction error is that after each feedback iteration, they drive changes in both *G* and *N*, and thus potentially result in faster learning. When *ϵ* = 0, the above model reduces to the AU model.

We now show that with these functions, the model can still encode expected reward and reward uncertainty. Subtracting the above two equations gives:

$$Q^{\left(t+1\right)}=Q^{\left(t\right)}+\alpha \left(\left(1+\epsilon \right)\left(r^{\left(t\right)}-Q^{\left(t\right)}\right)\right)-\beta Q^{\left(t\right)}$$ (23)

Hence at the stochastic fixed point:

$$Q^{*}=\frac{\alpha (1+\epsilon )}{\alpha (1+\epsilon )+\beta }E\left[r\right]$$ (24)

Thus the differences in the synaptic weights of D1 and D2 neurons encode scaled relative values of actions, which are also sufficient to choose the action with the highest value. Similarly adding Eqs (21) and (22) we obtain:

$$S^{\left(t+1\right)}=S^{\left(t\right)}+\alpha \left(\left(1-\epsilon \right)\left|r^{\left(t\right)}-Q^{\left(t\right)}\right|\right)-\beta S^{\left(t\right)}$$ (25)

Hence at the stochastic fixed point:

$$S^{*}=\frac{\alpha (1-\epsilon )}{\beta }E\left(\left|r-Q^{*}\right|\right)$$ (26)

Using the analysis applied earlier to the AU model, we see that the sum of the weights of D1 and D2 neurons encodes a scaled version of deviation of the reward, under analogous conditions to those for the AU model (i.e., *σ*_*r*_ is relatively high with respect to *μ*_*r*_, or *β* is relatively small with respect to *α*(1 + *ϵ*)). However, when *ϵ* > 0, the weights *G*^(*t*+1)^ or *N*^(*t*+1)^ computed from Eqs (21) and (22) may become negative, but negative synaptic weights are not allowed in the model, so the calculations of the fixed points above are only valid for *ϵ* sufficiently small so that *G*^(*t*+1)^ and *N*^(*t*+1)^ are not negative.

**Fig 7:**
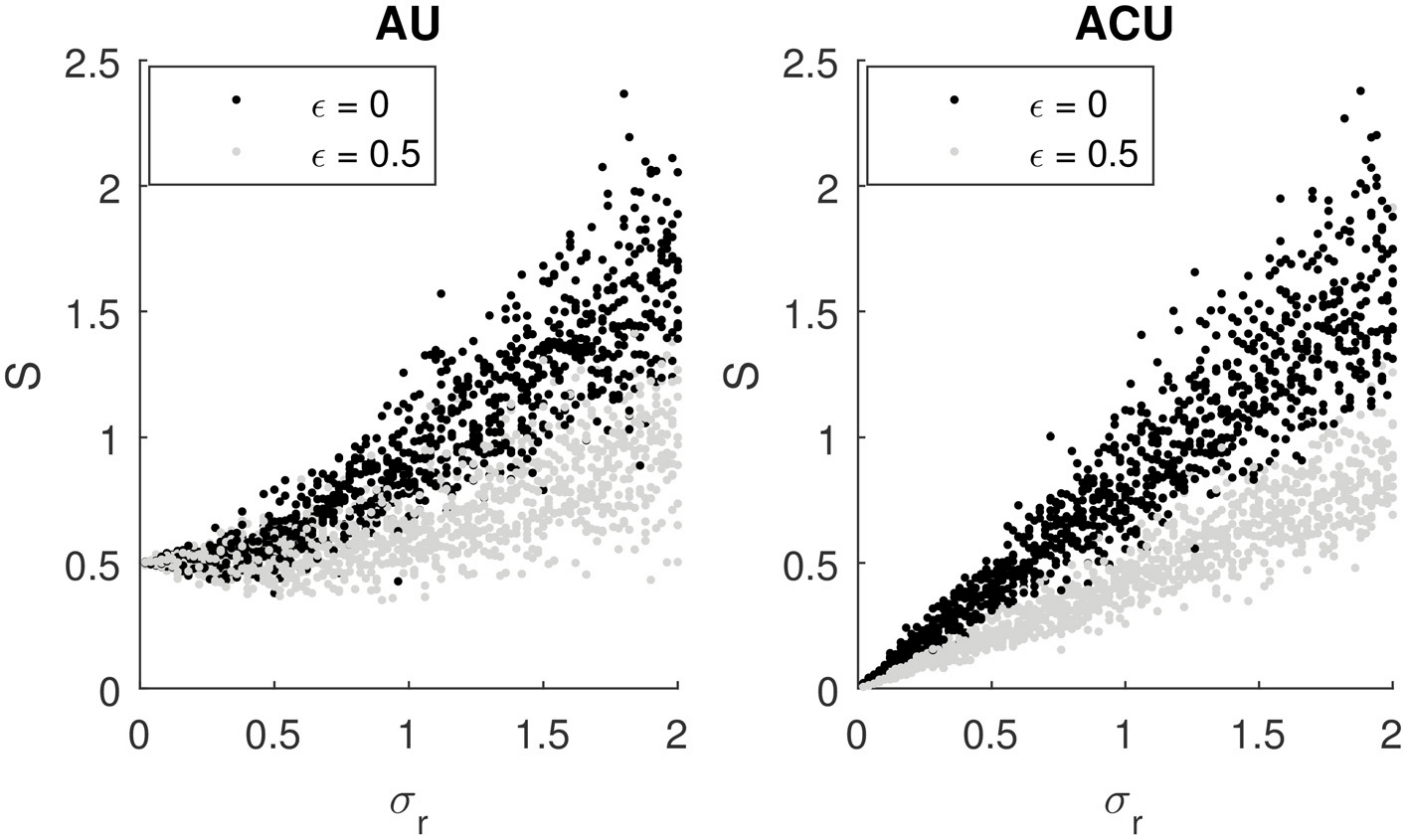
Comparison of the sum of weights in the Go and NoGo pathways (vertical axis) with the standard deviation of rewards (horizontal axis) in the original (black dots) and generalized (grey dots) versions of the AU (left panel) and ACU (right panel) models. In all simulations in this figure, *μ*_*r*_ = 1 and *α* = 0.1. The rewards were sampled from a Gaussian distribution. For each value of *σ*_*r*_ the model was simulated 10 times for 300 trials. For each simulation, the sum of *G* and *N* at the end of the simulation is displayed as a point on the figure. At the first trial of each simulation, the weights were initialized to *G* = *N* = 0.

To illustrate how this generalized AU model encodes reward uncertainty, the left panel in Fig 7 shows the results of simulations in the same setting as in Fig 3, but with a fixed value of *β* = 0.1, for different values of parameter *ϵ*. The figure shows that when *ϵ* = 0.5, the model also encodes reward uncertainty, but the encoding is less accurate than for *ϵ* = 0. In particular, when *S* is equal to a certain value, we can infer *σ*_*r*_ more precisely from the left panel in Fig 7 for *ϵ* = 0, as the range of *σ*_*r*_ resulting in the certain value of *S* is narrower for *ϵ* = 0 (e.g., *S* = 0.75 for *σ* ∈ [0.6, 1]) than for *ϵ* = 0.5 (e.g., *S* = 0.75 for *σ* ∈ [1, 2]).

### Learning reward uncertainty in actor-critic framework

In this section we show that the actor-critic model after small extension can learn both the mean and spread of rewards associated with actions. The model uses the same rule for the update of the critic (Eq (2)), and the plasticity of synapses of D1 and D2 neurons is described by equations similar to those for the AU model, but in which the prediction error is based on the reward estimated by the critic:

$$G_{i}^{\left(t+1\right)}=G_{i}^{\left(t\right)}+\alpha {\left|r^{\left(t\right)}-V^{\left(t\right)}\right|}_{+}-\alpha G_{i}^{\left(t\right)}$$ (27)

$$N_{i}^{\left(t+1\right)}=N_{i}^{\left(t\right)}+\alpha {\left|r^{\left(t\right)}-V^{\left(t\right)}\right|}_{-}-\alpha N_{i}^{\left(t\right)}$$ (28)

For simplicity, in the above equations we set the decay constant *β* = *α*, which will also allow relating the model to advantage learning [39, 40]. We will refer to a model with the actor described by the above equations, with the critic by the standard Rescorla-Wagner rule of Eq (2), and with the OpAL choice rule of Eq (6), as the Actor-Critic learning Uncertainty (ACU). We now show that the ACU model estimates both mean and spread of rewards associated with action *i*, which we denote by *μ*_*i*_ and *σ*_*i*_, respectively.

To see that the mean rewards are encoded in the difference between *G*_*i*_ and *N*_*i*_, we subtract the above equations, and using Eq (7), we obtain:

$$Q_{i}^{\left(t+1\right)}=Q_{i}^{\left(t\right)}+\alpha \left(r^{\left(t\right)}-V^{\left(t\right)}\right)-\alpha Q_{i}^{\left(t\right)}$$ (29)

This update rule differs from that of the original actor-critic model of Eq (3) in that it includes a decay term, and the rule is known as advantage learning [39, 40] (for reasons that will become apparent below). Let us now find the value the vicinity of which *Q*_*i*_ approaches, by noting that at the stochastic fixed point the following condition must hold:

$$E [\alpha \left(r-V^{*}\right)-\alpha Q_{i}^{*}]=0$$ (30)

Rearranging the terms in the above equation, we see:

$$Q_{i}^{*}=\mu_{i}-V^{*}$$ (31)

Namely, *Q*_*i*_ at the stochastic fixed point is equal to the expected reward for action *i* relative to the overall average reward in the current state (this quantity has been termed the advantage of action *i*). Note that knowing the relative values of the actions available in a given state is sufficient for selecting the action with the highest value. The value of the state V* is equal to the average value of all actions weighted by how frequently they are selected:

$$V^{*}=\sum_{i}P_{i}^{*}\mu_{i}$$ (32)

In this model, the sum of $G_{i}^{\left(t\right)}$ and $N_{i}^{\left(t\right)}$ also approximates reward uncertainty. Adding Eqs (27) and (28) we obtain:

$$S_{i}^{\left(t+1\right)}=S_{i}^{\left(t\right)}+\alpha \left|r^{\left(t\right)}-V^{\left(t\right)}\right|-\alpha S_{i}^{\left(t\right)}$$ (33)

At the stochastic fixed point, the expected change in the sum of weights should be equal to 0, hence:

$$E [\alpha \left|r-V^{*}\right|-\alpha S_{i}^{*}]=0$$ (34)

Rearranging terms, we see that the sum of weights *G*_*i*_ and *N*_*i*_ at the fixed point is:

$$S_{i}^{*}=E [\left|r-V^{*}\right|]$$ (35)

The above equation implies that when V* = *μ*_*i*_, the sum of *G*_*i*_ and *N*_*i*_ is equal to the deviation of the reward from the mean. We now consider three situations when V* is close to *μ*_*i*_.

First, when only one action is available, and chosen on all trials, then V* = *μ*_1_, and hence $S_{1}^{*}∼\sigma_{1}$. This property is illustrated in the right panel of Fig 7, where black dots show the uncertainty estimated by the ACU model in simulations with a single action. Note that *S* is proportional to reward uncertainty for the entire range of *σ*_*r*_, so with a single action, the ACU model can accurately encode uncertainty for a wider range of *σ*_*r*_ than the AU model (cf. black points in left and right panels of Fig 7).

Second, when a few actions *i* ∈ *I* have similar mean rewards, while other actions *j* ∈ *J* give much lower rewards, then *P*_*j* ∈ *J*_ are close to 0. In this case, V* is equal to a weighted average of *μ*_*i* ∈ *I*_, but since we assumed that all *μ*_*i* ∈ *I*_ are similar, then V* is close to *μ*_*i*_ for *i* ∈ *I*. Hence the ACU model estimates well the spread of reward distribution for actions with the highest mean reward, i.e., those most frequently selected. It may not estimate the spread of other actions, but this does not matter, as these actions are typically not selected anyway.

**Fig 8:**
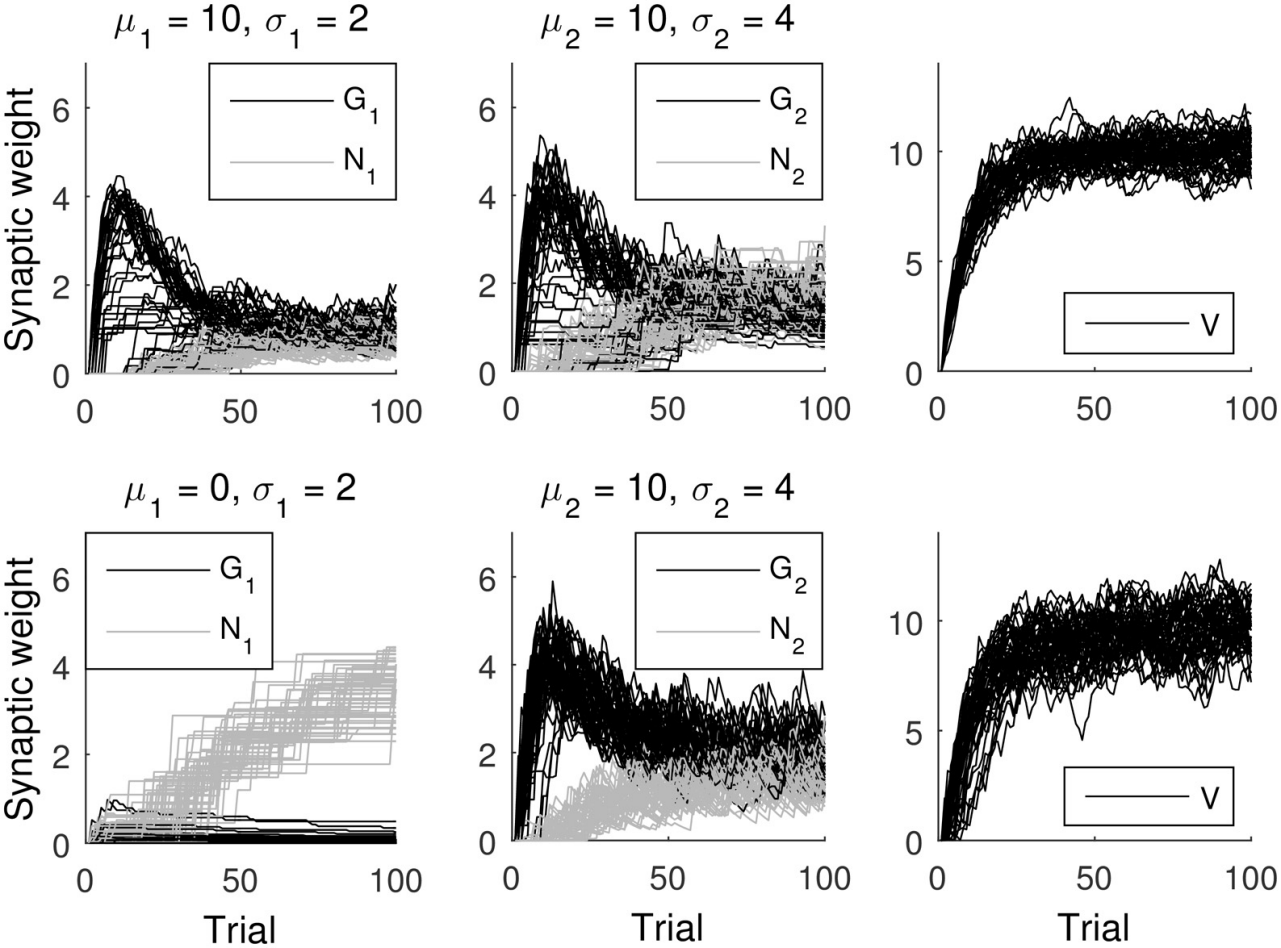
Changes in the variables of the ACU model simulated in a two-alternative choice task as a function of trial number. The rewards were sampled from a Gaussian distribution. Different rows correspond to simulations with different mean rewards *μ*_*i*_ (indicated above the panels), and different columns show: synaptic weights describing the tendency to select *G*_*i*_ and inhibit *N*_*i*_ for the two actions and the value of the state *V*. Standard deviations of reward *σ*_*i*_ associated with the two actions are indicated above the corresponding panels. Here, both *G* and *N* were initialized at 0, and we set *α* = 0.1 and the parameters of the choice rule to *a* = *b* = 1. For each of the panels, the simulation was run 50 times, for 100 trials each.

Different rows in Fig 8 show simulations of the ACU model for different reward distributions and illustrate changes in synaptic weights as learning progresses. In the first simulation, the two actions have the same mean reward, and it can be seen in the top row that the value *V* converges to the expected reward. For each action, *G*_*i*_ and *N*_*i*_ converge to values equal to each other, because the ACU model encodes in *G*_*i*_ − *N*_*i*_ the relative value of actions which are equal to 0 here. In the simulation, the second action has uncertainty twice as high as the first one, and indeed one can see in the top row of Fig 8 that *G*_2_ + *N*_2_ converges to a value twice as high as *G*_1_ + *N*_1_.

In the simulation illustrated in the bottom row of Fig 8, the first action has a smaller expected reward. The model learns to select the second action on a great majority of trials, which results in the expected reward *V* converging towards the mean reward of the second action. The model estimates well the deviation of rewards associated with the second action—note that *G*_2_ + *N*_2_ is similar in both rows of Fig 8. Finally, the model does not estimate well the deviation of reward of the first action, but this does not matter, as this action is very rarely selected.

Third, the ACU model can still estimate reward uncertainty for actions with lower mean rewards than other actions available, if the uncertainty is sufficiently large. To understand this property, it is helpful to rewrite Eq 35 as:

$$S_{i}^{*}=E [\left|\left(r-\mu_{i}\right)+\left(\mu_{i}-V^{*}\right)\right|]$$ (36)

When *σ*_*i*_ is sufficiently larger than |*μ*_*i*_ − V*|, the first term in the above equation will dominate over the second, and *S*_*i*_ will be more closely proportional to *σ*_*i*_.

In summary, the AU and ACU models differ in the conditions under which their ability to estimate reward uncertainty is limited. The AU model does not precisely estimate the reward uncertainty in situations where the standard deviation of rewards is small relative to their mean. The ACU model has a limited ability to estimate uncertainty only in a subset of these situations, i.e., when the reward uncertainty is small and additionally the mean value of the action is substantially lower than for other actions available in the corresponding state.

Finally, it is worth mentioning that the learning rule of the ACU model can be generalized as described in the previous subsection, such that the weights of the actor are modified according to Eqs 21 and 22 but with *Q* replaced by *V*. The grey dots in the right panel of Fig 7 show that the uncertainty estimated by such a generalized ACU model is still proportional to the true variability of rewards but is encoded less precisely than in the original ACU model. Furthermore, a simulation of the ACU model analogous to that shown in Fig 5 produced qualitatively similar behavior as the AU model; thus, an increased tendency to take risky options with a high level of DA is a general property of a class of models encoding reward uncertainty in *G* + *N*.

### Comparison with the OpAL model

**Fig 9:**
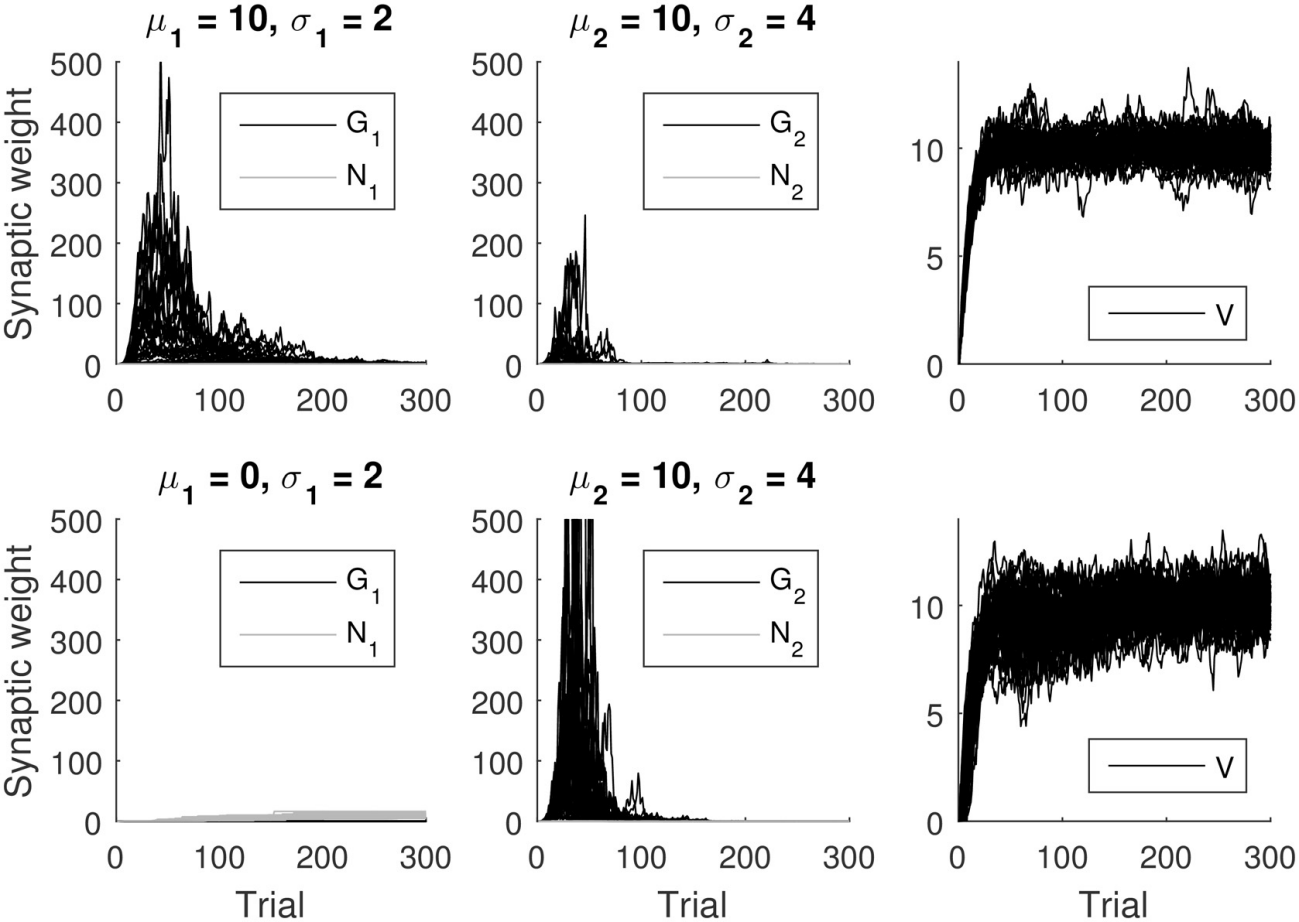
Changes in the variables of the OpAL model simulated in a two-alternative choice task as a function of trial number. The rewards were sampled from a Gaussian distribution. Different rows correspond to simulations with different mean rewards *μ*_*i*_ (indicated above the panels), and different columns show: synaptic weights describing the tendency to select *G*_*i*_ and inhibit *N*_*i*_ for the two actions and the value of the state *V*. Standard deviations of reward *σ*_*i*_ associated with the two actions are indicated above the corresponding panels. Here, both *G* and *N* were initialized at 0.1, and we set *α* = 0.1 and the parameters of the choice rule to *a* = *b* = 1. For each of the panels, the simulation was run 50 times, for 300 trials each.

We also investigated the behavior of the OpAL model [15] in the presence of reward uncertainty. Fig 9 shows simulations of the OpAL model in the same tasks used for the ACU model in Fig 8. Top rows of Fig 9 show simulations of a task in which the two actions have the same mean reward but differ in reward deviation. In the initial trials, in which the reward prediction error is positive, *G*_*i*_ increase exponentially. The exponential increase arises due to the multiplication of prediction error by *G* or *N* in Eqs 4 and 5, which results in a rate of weight changes that is proportional to the weights themselves. Once the reward prediction becomes equal to 0 on average, the weights start to decay towards 0. The weights have a stochastic fixed point at *G*_*i*_ = *N*_*i*_ = 0 in the OpAL model, because when *G*_*i*_ = *N*_*i*_ = 0, there are no changes in weights according to Eqs 4 and 5. In the task simulated in the top panel of Fig 9, this fixed point was attractive, and all weights of the actor eventually approached 0. It is interesting that this decay was faster for the option with higher uncertainty, as for this option the larger fluctuations in the reward prediction error drove the weights to the fixed point faster. In the task simulated in the bottom panel of Fig 9, this fixed point was attractive only for the action with the higher value, while for the other action, *N*_*i*_ increased with time.

It is evident from Fig 9 that the OpAL model does not encode reward uncertainty in the weights close to convergence, and the dynamics of weight changes is much more volatile than in the ACU model (note that the range of vertical axes in Fig 9 is two orders of magnitude higher than in Fig 8). Furthermore, when two actions have equal mean reward, as in the top panels of Fig 9, after extensive training, all weights *G*_*i*_ and *N*_*i*_ converge to 0, so the probability of choosing a more risky option becomes exactly 0.5, according to Eq 6, irrespective of the values of parameters *a* and *b*. Hence in this case, the probability of a risky choice predicted by the OpAL model is not dependent on the level of DA.

The OpAL model is able to capture the effects of dopaminergic medications seen in a series of experiments [14, 41, 42], which as we will see below, are challenging for the AU and ACU models. These experiments were designed to test the effects of DA on learning from positive and negative feedback, but in these studies the feedback uncertainty also varied between choice options. During these experiments the participants were presented with Japanese characters, were asked to choose one them, and subsequently received feedback indicating whether their choice was correct. For clarity, let us consider a simplified version of the task. Assume that during the training phase, the participant is presented on each trial with 3 letters which we will refer to as A, B and C. The probability of obtaining “Correct” feedback after selecting each of the 3 options is 0.8, 0.2 and 0.5 respectively. After the training, the participant is presented with a choice between A and C, or with a choice between B and C. The fraction of A vs. C trials in which the participant chooses A has been interpreted as a measure of learning from positive feedback (as stimulus A was associated with the highest probability of “Correct” feedback). Conversely, the fraction of B vs. C trials in which the participant does not choose B has been interpreted as a measure of learning from negative feedback (as stimulus B was associated with the highest probability of “Incorrect” feedback). It has been observed that Parkinson’s patients on dopaminergic medications exhibit higher accuracy in choosing A than in avoiding B, while the opposite pattern is present off medications [14]. Furthermore, it has been suggested that this effect is dependent on the medication state during testing rather than during encoding [42].

**Fig 10:**
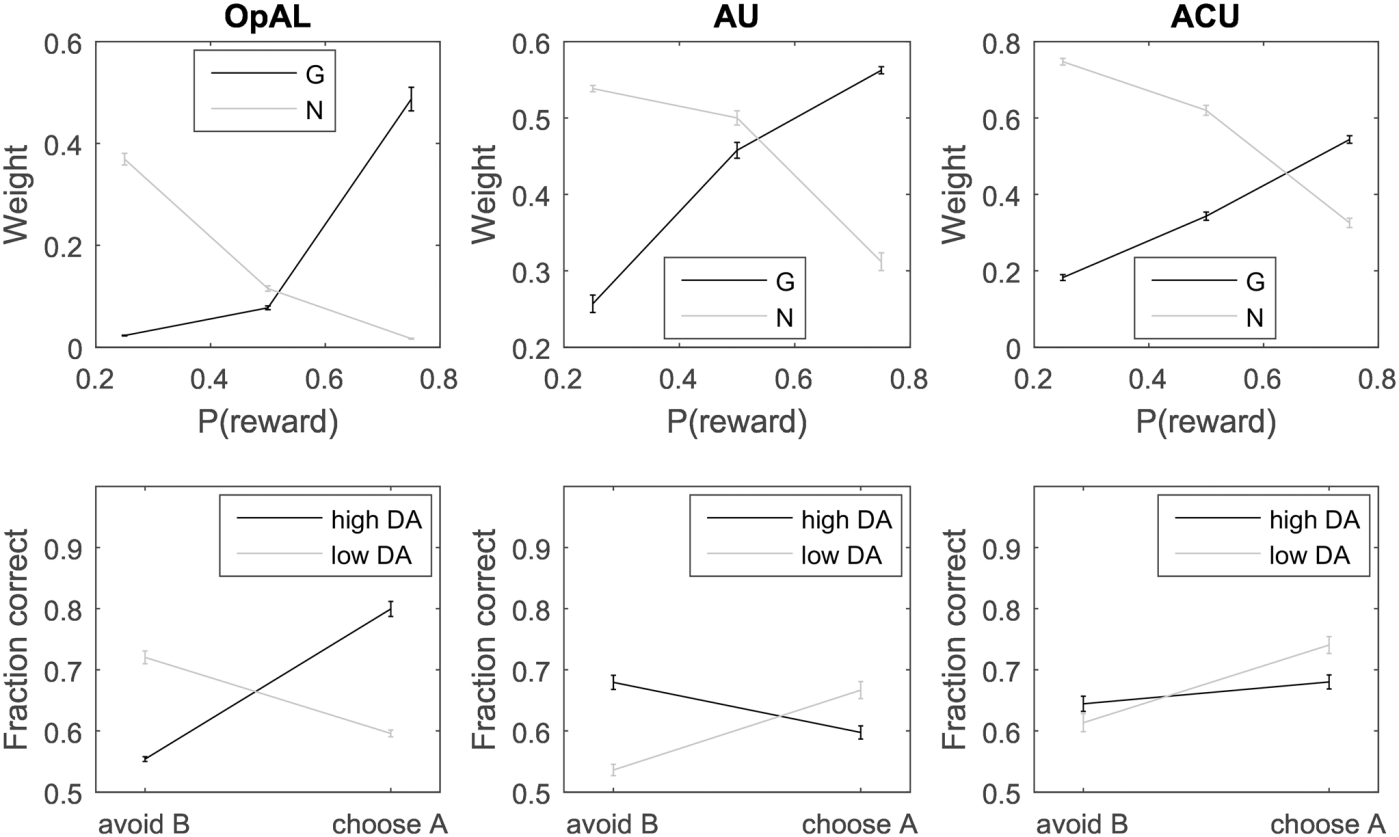
Comparison of the behavior of different models (labelled above columns of panels) in the Japanese letter learning task. The top panels show the weights at the end of the simulation, and the bottom panels the probability of choosing A and avoiding B (computed from Eq 6). At the start of each simulation *V*, *G* and *N* were initialized at 0.1, and we set *α* = *β* = 0.1. The parameters of the choice rule to were set to *a* = *b* = 2 during training, while during test they were set to *a* = 4, *b* = 0 in the simulated on medication condition, and to *a* = 0, *b* = 4 in the simulated off medication condition. For each of the models, 100 simulations were run, with 100 learning trials each, and error bars show standard error across simulations.

The OpAL model is able to replicate these effects [15]. While simulating learning in this task, we assumed that the model receives a reward of *r* = 1 when “Correct” feedback is given, and no reward *r* = 0 after “Incorrect” feedback. The top left panel in Fig 10 shows the weights learned by the OpAL model. As expected, *G*_*i*_ increase with the probability of reward, while *N*_*i*_ decrease. Importantly, the relationship between weights and reward probability is non-linear. This non-linearity arises from the multiplication of prediction error by *G*_*i*_ or *N*_*i*_ in Eqs 4 and 5, which as mentioned above, results in an exponential growth of the weights and thus magnification of weights with high values. The bottom right panel in Fig 10 illustrates how the values of the weights affect behavior during test. In the simulated on medication condition, the choice is primarily affected by weights *G*_*i*_ (Eq 6). Thus the accuracies in choosing A and avoiding B depend on |*G*_*A*_ − *G*_*C*_| and |*G*_*B*_ − *G*_*C*_|, respectively. Since |*G*_*A*_ − *G*_*C*_|> |*G*_*B*_ − *G*_*C*_| in the top left panel, the probability of choosing A is higher than the probability of avoiding B on medications in the bottom left panel. In the simulated off medication condition, the choice is primarily affected by weights *N*_*i*_, and hence the model is better at avoiding B than choosing A for analogous reasons. The choice pattern in the bottom left panel of Fig 10 is qualitatively consistent with that observed in experimental studies [14, 41, 42].

The top panel in the middle column of Fig 10 shows the weights learned by the AU model. Here also, *G*_*i*_ increase with reward probability, while *N*_*i*_ decrease. However, in the AU model the sum of weights *G*_*i*_ + *N*_*i*_ is highest for option C, which gives reward on 50% of trials and thus has highest reward variance. Consequently, the relationships between weights and reward probability are concave for the AU model, rather than convex as they were for the OpAL model. This results in the opposite effect of DA on choosing A and avoiding B relative to the OpAL model (cf. left and middle panels in the bottom row of Fig 10).

The right panels of Fig 10 illustrate that the behavior of the ACU model is qualitatively similar to that of the AU model. However, the predicted effect of medications on choice probability in ACU is smaller than in AU, because the relationships between weights and reward probability are more linear for ACU. This occurs because ACU estimates the deviation of reward from the mean across all trials (Eq 35) rather than from the mean reward for a given option, as in AU.

The OpAL model also described the dependence of learning rates *α* for *G*_*i*_ and *N*_*i*_ on the level of DA [15]. Simulations of the AU and ACU models indicate that increasing the learning rate for *G*_*i*_ (or *N*_*i*_) scales up the learned values of *G*_*i*_ (or *N*_*i*_) but does not change the convexity/concavity of the relationship between weights and reward probability, and hence does not change qualitatively the predicted effects of DA during testing on the probability of choosing A and avoiding B.

In summary, the simulations of the AU and ACU models produced qualitatively different patterns of effects of dopaminergic medications on choosing A and avoiding B than observed experimentally [14, 41, 42]. A critical feature of the OpAL model that allows it to capture the experimentally observed effects is the multiplication of prediction error by *G* or *N* in Eqs 4 and 5, but it is this very property that also caused unrealistically volatile weight changes in simulations of Fig 9.

It is interesting to ask under what assumptions the pattern of weights in the top left panel of Fig 10 (that allows reproducing the effects of medications on choosing A and avoiding B) could be obtained in a model learning reward uncertainty. In our simulations we assumed that “Correct” and “Incorrect” feedback were mapped on rewards of 1 and 0. However, it is unclear if the brain simply maps abstract feedback on the reward. It is possible that instead the brain infers that option C is unpredictable and does not engage in learning about it, which would result in relatively low *G*_*C*_ and *N*_*C*_, as in the top left panel of Fig 10. This interpretation together with the AU (or ACU) model predicts that if an actual (e.g., monetary) reward is given as feedback, the effect of dopaminergic medications on choosing A and avoiding B should reverse (or be very small). This interpretation is consistent with a result of experiments employing a modified version of the Japanese letter task with more salient feedback, i.e., smiling and sad faces, in which no effect of medications was found [43]. However, to fully test this interpretation, further studies are needed that could for example use explicit monetary reward.

## Discussion

In this paper we presented a class of models that can learn both the mean reward and reward uncertainty. The models describe how BG can control the influence of risk on choices and choose actions that not only maximize expected rewards but also minimize risks. Below we relate the models to experimental data, state further predictions, and discuss relationships with other computational models.

### Relationship to experimental data

We discuss here the relationships between predictions of the models and experimental data, including behavior and neural activity. Since in this paper we presented several models, it will be important to distinguish in the future which of them provide the best description of learning uncertainty in the basal ganglia. To differentiate between predictions specific to individual models and common to other models, we will use the term “the models” to refer to a class including all models introduced in this paper.

We already demonstrated in the Results section that the models account for the effect of pharmacological manipulations affecting dopaminergic receptors on risk aversion in reinforcement learning tasks in rats. The studies investigating the effect of DA on human decisions involving risks use two types of paradigms: one in which the mean and spread of rewards associated with choice options are explicitly described to the participant before each decision, and one in which they are gradually learned from feedback. Since human behavior is very different in these paradigms [44], and the models assume that the mean and deviation of rewards are learned in cortico-striatal synapses, below we only focus on studies involving learning from experience. The most commonly used paradigm in such tasks is the Iowa gambling task in which participants choose between decks of cards differing in reward variance. In agreement with the models, Parkinson’s patients receiving dopaminergic medications choose the risky decks more frequently than healthy controls, but this effect is not present in patients that have not been put on medications yet [45], or who stopped receiving medications [46].

The models introduced in this paper do not describe behavior in decision tasks in which information about risks associated with different options is explicitly presented before each trial. It is likely that processing information about uncertainty in such tasks involves different neural mechanisms and circuits than those learning about reward uncertainty over many trials.

The models are also consistent with the results of a recent study showing that optogenetic activation of striatal D2 neurons decreases the probability of choosing options with high reward variance [47]. Optogenetic activation of D2 neurons corresponds to a scenario illustrated in the bottom panel of Fig 4, where the choice is primarily driven by D2 neurons, and thus the risky option is inhibited.

The AU and ACU models differ in the predicted activity of DA neurons when the reward exactly matches the expected reward in tasks where only one action is available. In the ACU model, DA response is assumed to carry (*r* − *V*) where V* = *E*[*r*], so when *r* = *E*[*r*], DA neurons should not change their firing rate. By contrast, in the AU model the DA release is assumed to encode (*r* − *Q*) where Q* < *E*[*r*] (see Eq 13), so when *r* = *E*[*r*], DA neurons should increase their firing rate above baseline. Experimentally observed DA responses after expected rewards differed between experimental studies. For example, DA neurons were found to maintain their activity in classical conditioning in some studies [6, 7], while an increase was observed in others [48, 49]. Thus, more research is necessary to establish factors determining DA response to expected reward.

### Experimental predictions

The AU model predicts that learned synaptic weights in BG are insensitive to small standard deviations of reward; thus, it predicts that an individual’s choices are not affected by small enough uncertainty in reinforcement learning tasks. By contrast, the ACU model predicts that biases in estimation of reward uncertainty should only be present for actions with mean rewards much lower than those of other actions.

The models predict that overall activity in striatum should be higher during choice between options with high reward variance than during choice between options with lower reward variance but similar mean, because in the models the spread of rewards is encoded in *G*_*i*_ + *N*_*i*_, so higher reward variance should increase the activity of both D1 and D2 striatal neurons. This prediction could be easily tested using functional MRI.

The models predict that synaptic plasticity will depend on the current value of the weight itself (i.e., *G*_*i*_ or *N*_*i*_), because the weight update rules include decay terms proportional to the weights themselves. Thus the models predict that the stronger the weight of a synaptic connection, the higher the amplitude of induced long-term depression. Such dependence of plasticity on the value of weights has been observed in neocortex [50], and it would be very interesting to see if it is also present in cortico-striatal synapses.

### Relationship to other computational models

In addition to the models presented in this paper, reward uncertainty can be learned by a wide family of models in which the decay terms are proportional to the estimated uncertainty, and these models were analyzed in [28]. The models in this family can learn reward uncertainty even for small deviations. However, to implement such learning rules, the information about the uncertainty would need to be provided to a synapse, e.g., by a second neuromodulator. The models in this family predict that the release of this neuromodulator would need to be dependent on uncertainty and promote long-term depression of cortico-striatal synapses. Three different neuromodulators have been proposed to encode information about estimated (or expected) reward uncertainty: tonic DA [51], acetylcholine [52], and serotonin [30]. All of these neuromodulators have been shown to affect risky decisions [12, 53–55]. However, we have not found support in existing experimental data for predictions of our models employing multiple neuromodulators, hence we did not include them in this paper.

In previous reinforcement learning models that described learning about uncertainty [30, 56], the estimate of reward variance was updated on each trial proportionally to “variance prediction error”, which is equal to the difference between the square of reward prediction error and the current estimate of variance. An interesting model describing how such learning could be implemented in BG [57] suggested that the variance of rewards is encoded in striatal neurons co-expressing D1 and D2 receptors. This model assumed that such neurons could increase their weights both when the prediction error is highly positive (like D1 neurons) and when it is strongly negative (like D2 neurons). However, the neurons co-expressing D1 and D2 receptors form only a small proportion of striatal neurons [58], and the models we propose describe learning of reward deviation in the great majority of striatal projection neurons that express either D1 or D2 receptors.

An interesting reinforcement learning model has also been proposed in which choosing risky options can be avoided without explicitly learning the spread of reward distributions for different options [59]. In this model, the weight update rules are modified such that *Q*_*i*_ is decreased when action *i* leads to a reward with higher variance. This model is efficient when the desired level of risk aversion is known and fixed before the learning starts, but unlike the models presented in this paper, it does not allow the trained system to be easily switched from risk aversion to more neutral or risk seeking behavior.

Reward uncertainty is also likely to be estimated in the cortex. A particularly interesting model [60] describes how the variance of any feature of the stimulus (including reward) can be estimated in a neural circuit with organization similar to that of the neocortex, and it has been shown how this learning about variance can be implemented with local Hebbian plasticity [61]. It is highly likely that the mechanisms of learning uncertainty in neocortex and striatum can operate in parallel. Furthermore, these two structures may estimate complementary measures of dispersion: the cortical model [60, 61] estimates variance, while the models presented here estimate the mean absolute deviation (which is less affected by outliers).

In this paper we focused on one particular type of uncertainty associated with variable rewards in a stationary environment, which is typically called “expected uncertainty” [52]. But there is also another type of uncertainty connected with rapid changes (or volatility) of mean reward, referred to as “unexpected uncertainty” [52]. It is likely that there are complementary neural mechanisms which estimate unexpected uncertainty. For example, it has been proposed that striatal cholinergic tonically active interneurons detect changes in reward contingency and increase the learning rate following such changes [62]. Areas beyond BG can also be involved in this process, as the activity in other brain regions has been shown to track reward volatility [63] and volatility prediction errors [64].

Finally, let us discuss the relationship of the ACU model to advantage learning [39, 40]. As mentioned in the Results section, the ACU model estimates the mean reward using the advantage learning rule; thus, the ACU model also provides a description of how this abstract rule may be implemented in the BG circuit. The advantage model was originally introduced to reconcile reinforcement learning models with animals’ innate tendency to approach highly rewarding stimuli [39, 40]. The central feature of the advantage model (also inherited by the ACU model) is that as learning progresses, the value *V* represented by the critic approaches the value of the best action in the current state, while the advantage *Q*_*i*_ of this action approaches 0. This property describes a transition from an instrumental action selection to a stimulus-response habit, as in the trained state the action selection is implemented in the advantage model by the innate tendency to approach high value states [39, 40].

The ACU model has an analogous property that in the absence of reward uncertainty, *G*_*i*_ decreases towards 0 as learning progresses. Selection under such circumstances is primarily driven in the ACU model by D2 neurons, as suboptimal actions have large *N*_*i*_, and thus are inhibited. This agrees with a recent proposal of D2 neurons being critical for choosing among actions [65]. It would be possible to also include in the ACU model the tendency to approach high value states, by including additional terms in the softmax choice rule, as in [66].

In the advantage and ACU models, the actor encodes the mean reward relative to the overall reward in the current state (Eq (31)). So although the actor has the information necessary to choose which action is best in the current context, it does not know whether it is worth selecting it at all (e.g., whether any *μ*_*i*_ > 0). The information on whether it is worth making a movement in the given state (i.e., on the average value of actions chosen by the actor) is encoded in the critic. Thus the models suggest that patch neurons, which the critic has been mapped onto, should also be involved in movement initiation. It is intriguing that patch neurons project to the dopaminergic neurons [19], so one could ask whether they may communicate the information on the value of making a movement via dopaminergic neurons. This idea is consistent with DA controlling the vigor of movements [67].

## Supporting Information

S1 Text Relationship between mean absolute deviation and standard deviation. (PDF)
