# Supplementary material for: Learning Reward Uncertainty in the Basal Ganglia
Source: PLoS Comput Biol. 2016 Sep 2;12(9):e1005062. doi: 10.1371/journal.pcbi.1005062 (PMC5010205; doi:10.1371/journal.pcbi.1005062)
Supplement: S1 Text — (PDF) [file pcbi.1005062.s001.pdf]

S1 Text. Relationship between mean absolute deviation and standard deviation

Here we compute the value of $E[|r - \mu_r|]$ for the most commonly considered reward distribution and show that for a normal distribution of rewards, $E[|r-\mu_r|]$ is proportional to standard deviation, while for Bernoulli distribution, it is proportional to variance.

To simplify notation, while analyzing the case of normal distribution, without loss of generality, let us consider the case $\mu_i = 0$. Then:

$$\begin{aligned} E[|r - \mu_r|] &= 2\int_0^\infty r \frac{1}{\sqrt{2\pi}\sigma_r} \exp\left(-\frac{r^2}{2\sigma_r^2}\right) dr \\ &= -\frac{2\sigma_r}{\sqrt{2\pi}} \int_0^\infty -\frac{2r}{2\sigma_r^2} \exp\left(-\frac{r^2}{2\sigma_r^2}\right) dr \\ &= -\sqrt{\frac{2}{\pi}}\sigma_r \exp\left(-\frac{r^2}{2\sigma_r^2}\right)\Bigg|_0^\infty = \sqrt{\frac{2}{\pi}}\sigma_r \end{aligned} \quad (1)$$

Let us now consider a Bernoulli distribution where $r = 1$ with probability μ_r, and $r = 0$ with probability $1 - \mu_r$. For such a distribution:

$$E[|r - \mu_r|] = |1 - \mu_r|\mu_r + |-\mu_r|(1 - \mu_r) = 2\mu_r(1 - \mu_r) = 2\sigma_r^2 \quad (2)$$
